# Primary hydatid cyst of the fallopian tube: A case report

**Published:** 2014

**Authors:** Zeinab Nazari, Jila Torabizadeh

**Affiliations:** 1 Department of Gynecology, Imam Khomeini Hospital, Mazandaran University of Medical Sciences, Sari, Iran.; 2 Department of Pathology, Imam Khomeini Hospital, Mazandaran University of Medical Sciences, Sari, Iran.

**Keywords:** Fallopian tube, Echinococcus, granulosus, Hydatid cyst

## Abstract

***Background: ***Human hydatid disease is caused by echinococcus granulosus and has a global distribution. It mainly affects the liver, but can involve other organs. In this paper, we present a case of a primary hydatid cyst of the fallopian tube.

**Case presentation:** A 69-year-old (gravida 16, para 16 woman) with abdominal pain and urinary frequency was presented to the Gynecology Clinic of Imam Khomeini Hospital Sari, Iran, in September 2011. On physical examination, there was a nontender abdominal mass under umbilicus. The sonography of abdomen and pelvic showed a multiloculated mass with thick septation in right adnexa suggesting mucinous ovarian tumor, while the uterus, left adnex, liver, spleen and kidneys were all normal. The patient’s chest x-ray was normal. Serum tumor markers including CEA, CA125, αFP and βHCG were negative. An exploratory laparotomy was performed. There was a 20 cm firm elastic mass in the anterior surface of uterus originated from the right fallopian tube and was removed. Hydatid cyst was confirmed by pathological examination.

***Conclusion: ***Although primary hydatid cyst of genital tract is rare, in high prevalence countries it should be considered.

Echinococcsis is an infection caused by larval stage of Echinococcus granulosus, E. multilocularis, or E.vogeli in human. The disease may result from the development of expanding parasite cysts in visceral organs (1, 2). It does not usually have any symptom, in symptomatic patients, the site and size of the masses are important. Iran is an important endemic focus of cystic hydatid disease (3). In one report from Iran the prevalence of positive serology for this disease in general population was 4.8% (4). The hydatid cysts of granulosus tend to form in the liver up to 70% or lung up to 30% but may be found in any part of the body including the brain, heart and bones (2). The involvement of genital organs is very rare and may be seen in some cases of hydatid disease in the presence of involvement of other organs but pure involvement of genital organ is very uncommon. When disease is found in reproductive organs, the uterus and ovaries are the more common sites than the cervix and fallopian tube. Pure tubal infection without involvement of other organs is very rare and only one reported case is found in the medical literature (5). In this study, we present a case of primary fallopian tube hydatid cyst without any evidence of disease in the other organs.

## Case Presentation

A 69-year old (gravida 16 para 16 woman) with abdominal pain and urinary frequency presented to the Gynecology Clinic of Imam Khomeini Hospital Sari, Iran, in September 2011.

She was from a rural area of south Khorasan province. On physical examination, there was a nontender abdominal mass under the umbilicus, although the liver and spleen were within normal size. She was admitted in gynecology ward and workup of abdominal mass was performed. 

The sonography of abdomen and pelvic showed a multiluculated mass with thick septation in the right adnexa suggesting mucinous ovarian tumor, while the uterus, left adnex, liver, spleen and kidneys were all normal. The patient’s chest x-ray was normal. Serum tumor markers including CEA, CA125, αFP and βHCG were negative. All other lab tests were in normal range except mild anemia.

**Figure 1:**
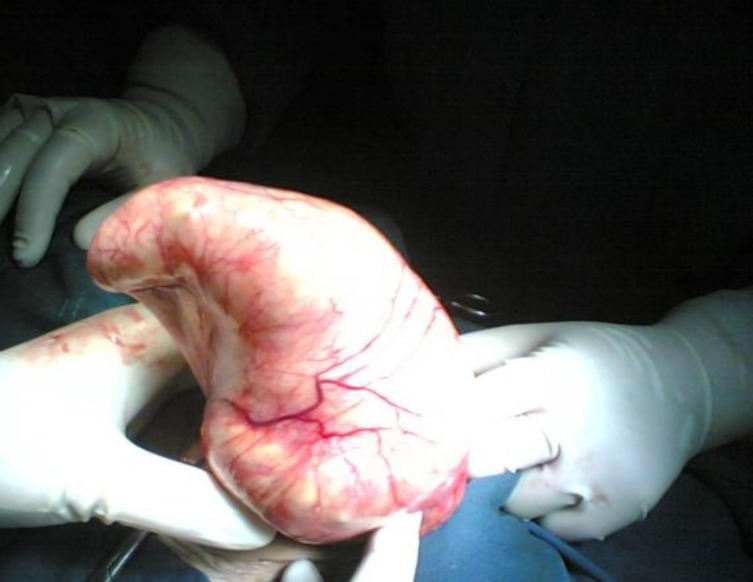
Anterior view of the mass during operation

**Figure 2:**
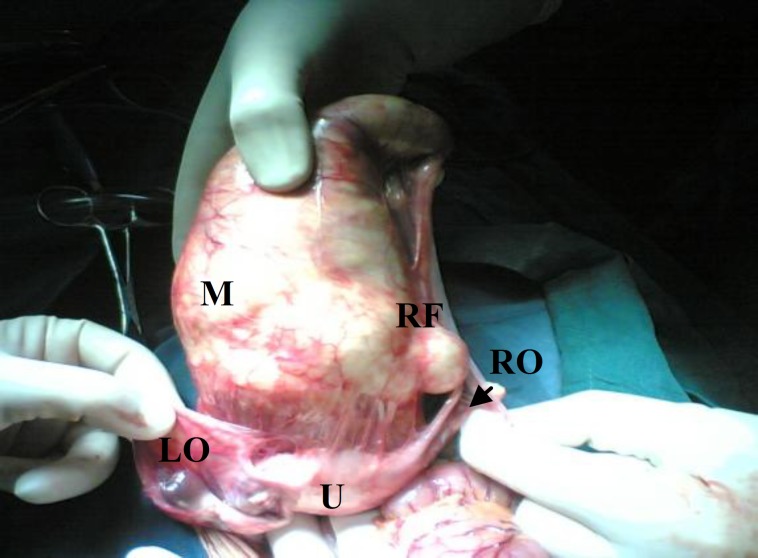
Posterior view of the mass during operation

An exploratory laparotomy with midline incision was performed, no ascites was present. There was a 20-cm firm elastic mass in the anterior surface of uterus originated from the right fallopian tube. Two ovaries were atrophic and normal (figures 1, 2). 

M=Mass, U=Uterus, RO= Right Ovary, LO= Left Ovary, RF= Right Fallopean Tube

**Figure 3:**
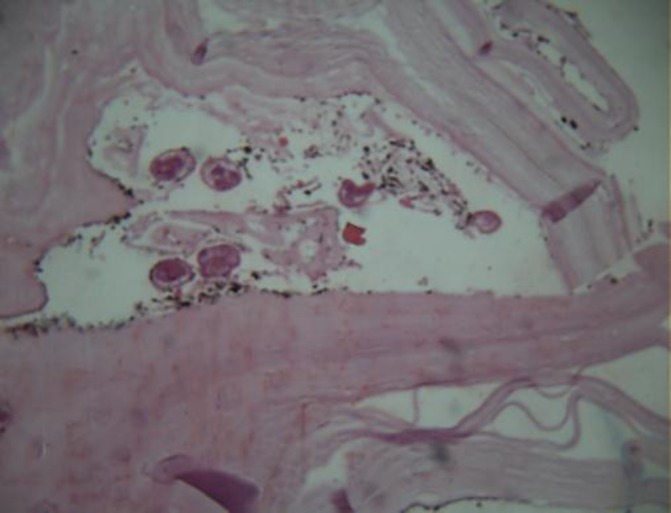
Portion of hydatid cyst (X10)

Exploration of entire peritoneal surface and organs were normal**. **She underwent total abdominal hysterectomy and bilateral salpingoophorectomy. Pathologic report confirmed the diagnosis of hydatid cyst (figure 3). The patient then referred for medical treatment. Postoperative follow-up was done for six months. After the surgery, she was in good condition.

## Discussion

Primary hydatid disease in reproductive organs is rare. Reviewing the literature, we found less than ten articles about primary involvement of reproductive organs, among them only in one article, the primary involvement of fallopian tubes was reported (5). The involvement of other organs such as liver, uterus, spleen and fallopian tubes have been demonstrated (6, 7). In fallopian tube, the disease presented as a paraovarian cyst (8). Fabre et al., reported that the disease is limited to ovary and fallopian tube (9). Primary ovarian hydatid disease has also been shown in the report of Adewunmi et al. (10). Other reports of genital tract hydatid cyst were pelvic cyst in Douglas pouch adherent to adnexa and surrounding structures (11). In one report from India, there was a primary hydatid cyst of uterine cervix and parametrium (12).

In this paper, we report the second case of primary fallopian tube hydatid cyst worldwide. This suggests that hydatid disease is still a health care problem in Iran, however, the atypical site of the cyst makes it difficult to diagnose. 

## Notes

**Conflict Interest: **There is not any conflict of interest for this study.
